# An analysis of heavy silicone oil treatment for inferior proliferative vitreoretinopathy

**DOI:** 10.1186/s12886-024-03834-7

**Published:** 2025-01-22

**Authors:** Maximilian Hammer, Amanda Ie, Katharina Eibenberger, Gerd Uwe Auffarth, Kanmin Xue

**Affiliations:** 1 Oxford Eye Hospital, Oxford University Hospitals NHS Foundation Trust, Oxford, UK; 2 Department of Ophthalmology and David J Apple Laboratory for Vision Research, Heidelberg University Hospital, Heidelberg, Germany; 3 Nuffield Laboratory of Ophthalmology, Nuffield Department of Clinical Neurosciences, University of Oxford, Oxford, UK; 4 Great Ormond Street Hospital for Children NHS Foundation Trust, London, UK; 5 Nuffield Department of Clinical Neurosciences, University of Oxford, Level 6 West Wing, John Radcliffe Hospital, Headley Way, Oxford, OX3 9DU UK

**Keywords:** Vitreoretinal surgery, Retinal detachment, Silicone oil, Endotamponade, Proliferative vitreoretinopathy, PVR

## Abstract

**Purpose:**

Proliferative vitreoretinopathy (PVR) is a complication of retinal detachment which requires multiple vitreoretinal surgical interventions and frequent use of oil endotamponade. In this study, we conducted an in-depth analysis of complications associated with the use of heavy silicone oil in the management of inferior PVR.

**Methods:**

A retrospective cohort study of 20 eyes that underwent vitrectomy for inferior PVR with use of heavy silicone oil (Densiron 68) between March 2021 and October 2022 at Oxford Eye Hospital. Complications were classified into major categories relating to intraocular pressure, inflammation, lens, and oil emulsification/migration. Visual outcomes and surgical success rate were also evaluated.

**Results:**

Fill-induced pressure spikes (> 30 mmHg) within 14 days post-surgery were common after Densiron tamponade, especially in patients previously on glaucoma drops. The number of glaucoma drugs were increased in 45% of patients during Densiron tamponade. In 20% of cases, an increased medication was continued long-term after Densiron removal. Significant cataract progression occurred in all phakic patients. In 25% of pseudophakic cases, posterior capsule opacification was noted. Inflammatory complications, such as anterior uveitis, were rare and any cystoid macular oedema was transient. No unexplained acute loss of vision following Densiron removal was encountered. The anatomical success rate at 30 days after Densiron removal was 70%. The mean (± SD) best-corrected visual acuities were 1.04 (± 0.79), 0.85 (± 0.62) and 0.50 (± 0.51) logMAR prior, during and after Densiron tamponade, respectively.

**Conclusion:**

The outcomes in this cohort treated with Densiron 68 were comparable to previously reported anatomical and functional results in cases with inferior PVR. IOP and lens-related complications require additional treatment during or after Densiron tamponade. Inflammatory complications rarely occurred over tamponade durations of around three months.

**Trial registration:**

Analyses were conducted as an internal quality improvement audit and as such did not require external IRB review.

## Background

Proliferative vitreoretinopathy (PVR) is the major cause for failure of retinal detachment surgery and occurs in 5–10% of cases [1]. Due to the effects of gravity, PVR membranes most frequently develop over the inferior retina [2]. Treatment is based on surgical revision with removal (delamination) of the tractional PVR membranes and allowing time for the fibro-proliferative response to subside [3]. Often at the end of the surgery for PVR-related redetachment, the vitreous cavity is filled with silicone oil.

The use of silicone oil in vitreoretinal surgery was first described by Cibis [4] and Armaly [5] in 1962. Lighter-than-water silicone oil creates an upward force, which is ideally suited to tamponading superior retinal breaks or giant retinal tears, although breaks in the inferior retina may be covered through strict posturing of the patient on the side. Over the past decades, the mechanical and surgical properties of silicone oil have been optimized to deal with a broader range of scenarios in retinal surgery. One of the major developments has been the creation of heavier-than-water silicone oil to better manage inferior pathologies. This is achieved by adding F6H8, a high density semifluorinated alkane, to lighter-than-water silicone oils consisting of polydimethylsiloxane-chains (PDMS). The greater density of heavy oil creates a downward force, which is better suited for treating inferior breaks and PVR. This modification, however, does not only influence the oil’s density but also its surgical behaviour (e.g. injection and removal technique) and biological properties [6–10].

The use of heavy silicone oil has greatly facilitated the management of complex retinal detachment and appears to be associated with a favorable safety profile [11]. So far, complications associated with heavy oil have mostly been reported in routine surgical patient cohorts such as primary retinal detachment repair [11–13]. Thus, it is unclear what rates of complications may occur in complex PVR management, or indeed in comparison with lighter-than-water silicone oils. Here, we conduct an in-depth retrospective analysis of all adverse effects associated with the use of Densiron 68 (Fluoron GmbH, Ulm, Germany) in the management of inferior PVR redetachments at a tertiary referral centre. Surgical outcomes were reported along with complications which were classified into four main categories: (i) intraocular pressure (IOP)-related, (ii) inflammatory, (iii) lens-related, and (iv) oil emulsification/migration.

## Methods

### Study cohort and ethical approval

This study is a retrospective, single-centre cohort study. Patients with inferior retinal detachment due to inferior proliferative vitreoretinopathy that underwent Densiron 68 removal between March 2021 and October 2022 at the Oxford Eye Hospital were included in this study. Analyses were approved by the Oxford University Hospitals Integrated Governance System (Oxford University Hospitals Quality Improvement Project approval no. 9440) and were conducted as an internal quality improvement audit. Informed consent was waived by the ethics committee and thus no informed consent was obtained.

### Surgical technique

Complete removal of Densiron 68 with its higher-than-water density requires technical modifications compared with lighter-than-water silicone oils. All patients in this study underwent Densiron removal using 23G viscous fluid extraction tip (VFC Pak, Alcon Constellation) and high vacuum to lift the Densiron bubble towards the nozzle tip. For this approach, three 23G trocars are placed at pars plana to allow for direct visualization using a light pipe, infusion and VFC extraction tip. In cases of significant posterior capsule opacification, a surgical capsulotomy was performed after Densiron removal. During the initial surgery, the vast majority of cases received localized PVR membrane peeling to alleviate traction (17 of 20 cases, 85%). Only one (5%) case received a focal retinectomy in order to flatten the retina as the PVR membrane could not be peeled.

### Exposure and outcome measures

Data were collected from electronic medical records comprising patient, surgery and outcome-related data. Spectral-domain OCT data were also assessed if available by experienced clinicians. All OCTs were acquired with a Spectralis (Heidelberg Engineering, Heidelberg, Germany). PVR was graded based on fundus images and clinical notes.

Every patient was assessed at 3 timepoints; prior, during and after Densiron tamponade. Complications and clinical outcomes were divided into 5 categories.

1. Inflammatory complications, including abnormal postoperative conjunctival injection, anterior chamber cells, cystoid macular oedema and choroidal thickness (subset with available SD-OCTs).
2. IOP-related complications, including the need for an increase in glaucoma medication during and after Densiron implantation as well as postoperative pressure spikes > 30 mmHg within the first 14 days after implantation.
3. Lens-related complications: rate and grade of cataract formation following Densiron tamponade, rate of combined cataract surgery at Densiron removal, rate of posterior capsule opacification in pseudophakic eyes at Densiron removal.
4. Emulsification-related complications, including the presence of emulsified oil droplets in the anterior chamber, migration of a greater oil volume to the anterior chamber, oil adhesion to the crystalline lens or the IOL and the need for anterior chamber washouts.
5. Outcomes: best-corrected visual acuity in logMAR and success rate of detachment repair.

Further, the incidence of the Removal of Silicone Oil (ROSO) syndrome, defined as otherwise unexplained reduction in visual acuity or scotoma after silicone oil removal, was analyzed.

### Visual acuity and final anatomical success rate

Hand movement and counting fingers were converted to logMAR using a previously used transcription used by the National Ophthalmology Database (NOD) [14, 15]. Counting fingers was thus equivalent to logMAR 2.1, hand movement to 2.4 and light perception to 2.7. Final success was defined as flat retina with no tamponade or further intervention needed at least 30 days after Densiron removal.

### Statistical analysis

Normal distribution was examined using the Kolmogorov-Smirnov test. Paired t-tests, one-way ANOVA or Wilcoxon-tests were conducted, as appropriate. P-values of < 0.05 were considered statistically significant. PRISM 8 (GraphPad Inc, USA) and Stata 17 BE (StataCorp, USA) were used for statistical analysis. Regarding choroidal thickness, only subjects with data available for both compared time points were included.

## Results

### Demographics

Twenty eyes of 20 patients were included in the study. Table 1 presents the patient characteristics. Patients were aged 63 ± 14 (SD) years and 80% male. The median duration of Densiron tamponade was 112 days (interquartile range, IQR: 101–137 days). The median follow-up duration after Densiron removal was 178 days (IQR: 104–399). Densiron 68 was implanted during the 2nd retinal surgery in 10 of the 20 (50%) cases. Three cases (15%) were implanted with Densiron 68 at primary detachment repair, the remaining cases were implanted with Densiron in their 3rd (5 cases, 25%), 4th (1 case, 5%) and 6th (1 case, 5%) retinal surgery. PVR was graded in accordance to Machemer et al. 1991, with 11 (55%) eyes having PVR grade C and the remaining 9 (45%) eyes having PVR grade B [16]. Seven of the 20 eyes (35%) were ‘macula on’ at the time of Densiron implantation (i.e. the macula had never been detached in the past).

**Table 1:** Patient characteristics

**Baseline characteristics**	
Age (mean ± SD)	63 ± 14
Male gender, n (%)	16 (80%)
Follow up (median, IQR), days	178 (104–399)
Duration of Densiron tamponade (median, IQR), days	112 (101–137)
Right eye (%)	8 (40%)
**Ocular comorbidities**	
Glaucoma under topical treatment, n (%)	3 (15%)
Number of previous surgeries (median, IQR)	2 (2,3)
**Previous tamponades**	
None (primary retinal detachment surgery)	3 (15%)
Gas	10 (50%)
Lighter-than-water silicone oil	4 (20%)
Gas and lighter-than-water silicone oil	3 (15%)
**Lens status at the time of Densiron implantation**	
Aphakic	1 (5%)
Phakic	7 (35%)
Pseudophakic	12 (60%)
**Macula status at the time of Densiron implantation**	
Macula on (%)	7 (35%)
Macula off (%)	13 (65%)

### Inflammatory complications

Cells in the anterior chamber were only noted in one individual during Densiron tamponade (5% of all cases), which resolved after Densiron removal. One patient developed conjunctival hyperemia due to subconjunctival Densiron droplets which faded after removal of Densiron and conjunctival wash-out. Cystoid macular oedema (CMO) was observed by OCT in 4 (20%) cases during Densiron tamponade. After Densiron removal, only two cases (10%) showed persistent CMO. One of these two cases slowly resolved over 8 weeks with topical corticosteroid therapy. The other case demonstrated persistent CMO (by 4 and 12 weeks after Densiron removal) associated with a suspected subretinal oil bubble. In all cases, prostaglandin analogues for raised intraocular pressure were excluded as potential causative agent for the CMO. CMO did not appear to be significantly associated with longer duration of intraocular Densiron 68 tamponade (*p* = 0.21).

### Choroidal thickness

**Fig. 1:**
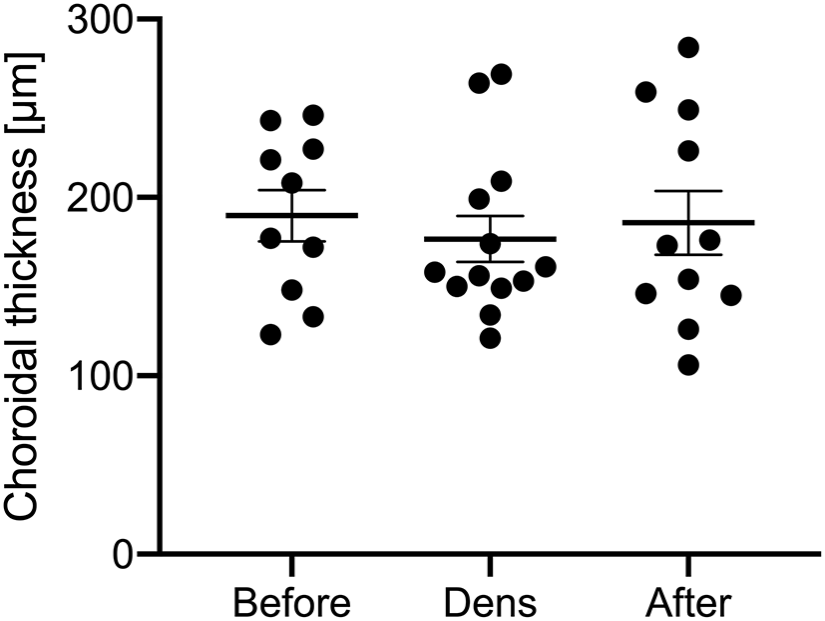
Submacular choroidal thickness before, during and after Densiron tamponade analyzed using enhanced depth optical coherence tomography (EDI-OCT). No significant changes in choroidal thickness were found. In particular, no significant choroidal thinning occurred, in contrast to previous reports with lighter-than-water silicone oil [54]

It was proposed that silicone oil may have an impact on the structure and function of the choroid. Thus, measurements of choroidal thickness by OCT may be clinically useful for monitoring chorioretinal health and deciding the timing of silicone oil removal in order to reduce the risk of retinal toxicity. Choroidal thickness was assessed in patients with sufficient enhanced depth-OCT quality for measuring choroidal thickness (before implantation of Densiron – 10 eyes; during Densiron tamponade – 13 eyes; and after Densiron removal – 11 eyes). We found no significant change in choroidal thickness over the course of Densiron tamponade in 10 eyes with available data at all time points (paired t-tests between before Densiron tamponade and after Densiron removal, *p* > 0.1). Figure 1 depicts the individual choroidal thickness measurements.

### Complications related to intraocular pressure

#### Postoperative IOP rise

Ten (50%) patients experienced a significant intraocular pressure rise (defined as IOP > 30 mmHg) during the first 14 postoperative days after Densiron implantation. All patients (3 of 3) that were on glaucoma drops prior to Densiron implantation experienced a significant intraocular pressure rise. One (5%) case presented with an IOP between 21 and 30 mmHg during the first 14 postoperative days after Densiron implantation.

### Increased glaucoma treatment during and after Densiron tamponade

**Fig. 2:**
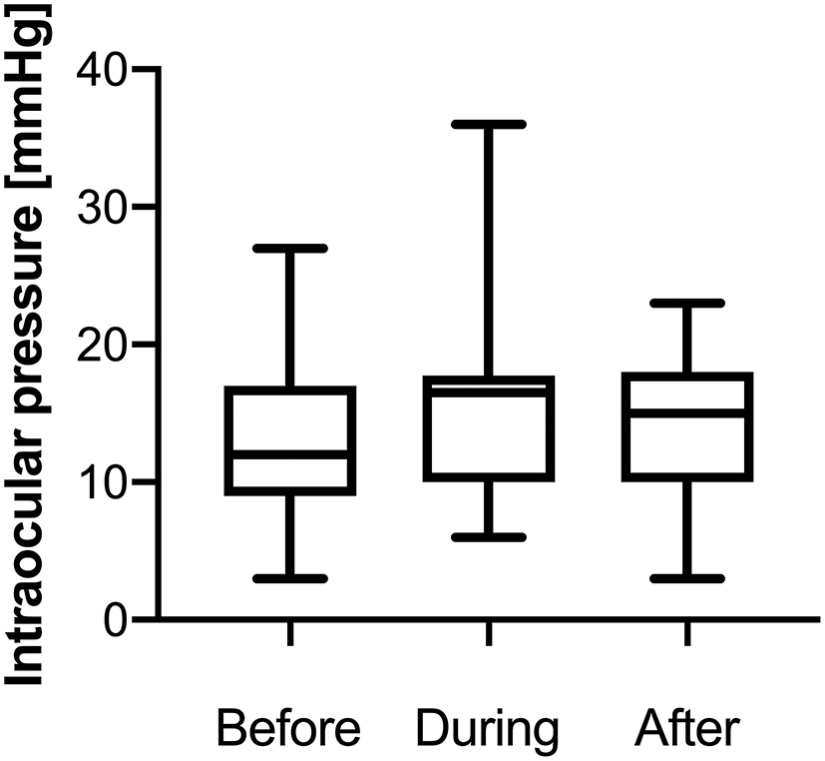
Intraocular pressure (IOP) before, during and after Densiron tamponade. IOP rises within 14 days after Densiron implantation were common, but controllable with topical glaucoma therapy. As a result, there was no significant overall difference in IOP during and after the use of Densiron compared with preoperative values

**Fig. 3:**
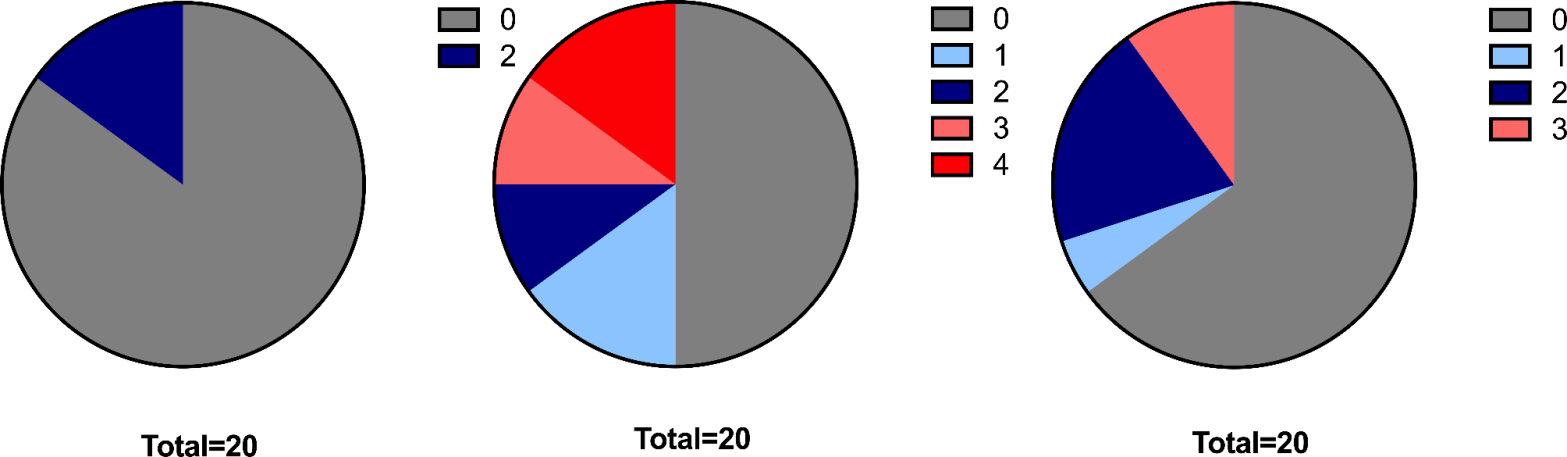
Number of glaucoma medications before, during and after Densiron tamponade. The pie charts present the number of eyes and number of topical glaucoma drops needed before (left), during (middle) and after (right) the use of Densiron. During Densiron tamponade, the use of topical glaucoma treatment increased. After Densiron removal, a reduction in the number of glaucoma drops was seen, but 4 of 20 eyes remained on an escalated glaucoma treatment regime compared with before Densiron tamponade

Nine (45%) patients were started on new glaucoma medications during Densiron tamponade. After Densiron removal, 4 patients (20%) with no previous history of glaucoma were kept on an increased number of glaucoma medications. Figure 2 shows the mean IOP ± SD of all patients before (12.4 ± 5.4 mmHg), during (15.0 ± 6.4 mmHg) and after (13.6 ± 5.3 mmHg) Densiron tamponade. Figure 3 depicts the number of patients on a different number of glaucoma medications. The number of glaucoma drops was increased during Densiron tamponade in approximately half of the study cohort: median (IQR) of 0 (0–0), 0.5 (0-2.5) and 0 (0–2) agents before, during and after Densiron tamponade, respectively. After Densiron removal, glaucoma treatment was reduced for most patients, but 4 (20%) eyes remained on a long-term higher topical regime when compared to the preoperative state. Adequate IOP control was achieved in all cases with medication, as indicated by no significant change in overall IOP prior, during and after Densiron tamponade (One-way ANOVA, *p* = 0.23). No patient required trabeculectomy or shunt surgery for refractory glaucoma.

### Lens complications

During Densiron tamponade, 7 (35%) patients were phakic, 12 (60%) were pseudophakic, and one (5%) was aphakic. All (7 of 7) phakic eyes showed significant cataract at time of Densiron removal warranting combined cataract surgery: graded as NUC Standard 2 (5 of 7 eyes), posterior subcapsular cataract grade 2+ (1 of 7 eyes), or both (1 of 7 eyes graded NUC Standard 3 and subcapsular cataract grade 2+). Three (25%) pseudophakic patients developed significant posterior capsule opacification at the time of Densiron removal. None of the pseudophakic patients had previously undergone capsulotomy.

### Direct Densiron-related complications

Prior to Densiron removal, emulsified oil droplets were only seen in the anterior chamber in one (5%) patient who had previously also received lighter-than-water silicone oil. In one patient (5%) a greater volume of Densiron migrated into the anterior chamber, associated with raised IOP and transient corneal oedema. This patient had no known risk factor predisposing to oil migration such as zonular defect or hypotony. A superior YAG iridotomy did not relieve the situation, thus anterior chamber washout was performed to facilitate continued Densiron tamponade of the retina. After Densiron removal, emulsified oil droplets were present in the anterior chamber in 3 out of 20 (15%) eyes. In one (5%) case, emulsified Densiron droplets adhered to the posterior surface of the IOL, but the patient was not visually impaired by the droplets and did not require further interventions. Two (10%) eyes received a separate anterior chamber washout due to retained oil droplets. No corneal complications requiring lamellar or penetrating keratoplasty were observed. No patient developed ‘removal of silicone oil’ (ROSO) syndrome.

### Visual outcomes and surgical success

**Fig. 4:**
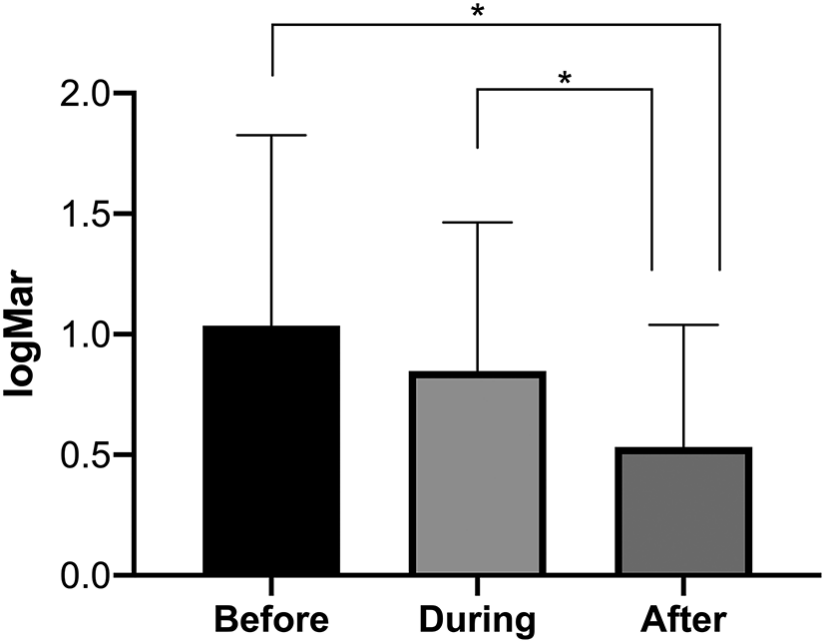
Best-corrected visual acuity (BCVA) before (20 eyes), during (20 eyes) and after (18 eyes) Densiron tamponade for the treatment of retinal detachment associated with inferior proliferative vitreoretinopathy. BCVA in logMAR. A significant increase in BCVA was observed. Mean and standard deviation are presented. * indicates *p* < 0.05

Visual acuity significantly improved at the final follow-up timepoint as shown in Figure 4. The mean (± SD) best-corrected visual acuities were 1.04 ± 0.79, 0.85 ± 0.62 and 0.50 ± 0.51 logMAR prior, during and after Densiron tamponade, respectively. Of note, seven (35%) out of 20 eyes were considered ‘macula on’ at the time of Densiron insertion. Final anatomical success rate was 70%, defined as retina fully attached without intraocular tamponade at the final follow-up timepoint (at least 30 days post-surgery). All retinal redetachments occurred after the removal of Densiron. All cases that did not achieve anatomical success at the last assessment timepoint subsequently underwent further interventions: 4 (20%) cases underwent one more vitrectomy with PVR peeling for recurrence of PVR-related redetachment; 2 (10%) cases underwent two more vitrectomies for the same indication. For these cases that required further surgery, the last recorded visual acuity prior to the occurrence of redetachment was used in the analysis.

## Discussion

Heavy silicone oil is a useful surgical adjunct for the management of retinal detachments complicated by inferior PVR. Until now, Densiron-related complications were either not analyzed in-depth or in less challenging cases such as primary inferior retinal detachment surgery. In this study, we present an in-depth analysis of 20 eyes with complex retinal detachments associated with inferior PVR managed with Densiron 68 tamponade. We divided possible complications into inflammation, IOP, lens and emulsification-related complications and evaluated visual outcomes after PVR repair. IOP and lens-related complications were by far the most common reason requiring intervention during Densiron tamponade and after Densiron removal.

### Intraocular pressure

Densiron-induced increase in IOP has been previously described in primary inferior retinal detachment surgery and, in particular, in comparison to lighter-than-water silicone oils. First, Wong et al. 2009 [12] noted an elevated IOP within 14 days after surgery, while no more significant difference was noted at 4 weeks postoperatively when compared to lighter-than-water silicone oil. Romano et al. in 2010 [13] found no significant difference in IOP between oil types in primary inferior retinal detachments. They reported that at day 1 postoperatively 20% of Densiron-filled eyes had an IOP of 25 mmHg or higher. In a recent study, Moussa et al. 2022 [17] found no difference in the rate of glaucoma surgery after lighter-than-water and heavier-than-water silicone oil endotamponade. Available data on IOP and heavy silicone oil in PVR cases is sparse, however two observations have been previously mentioned: (i) Densiron-related pressure spikes occur mostly within days after surgery and are common [18–23]; (ii) persistent elevated IOP after removal of the heavy silicone oil can occur in up to a quarter of cases [18, 24]. Most of the previous reports mention that IOP could be controlled with topical glaucoma medication, but have not elaborated on the number or duration of the glaucoma medications [25]. In our study, no overall statistically significant difference in IOP was found when comparing before, during and after Densiron tamponade (*p* = 0.23), suggesting adequate IOP control. However, the management of IOP was complex and required escalation of glaucomatous medication in many cases. In line with previous literature, all IOP spikes occurred within 14 days after surgery and could be topically controlled. It is important to note that the majority of patients in the cohort received four weeks of QDS 0.1% dexamethasone drops as part of the standard post-vitrectomy medication regime. As this patient cohort underwent multiple surgeries and received topical steroid regimes multiple times, a more rapid steroid response than usual is possible which would confound the IOP spike rate observed. Previous studies in PVR cases did not report the use of steroid or alternative non-steroidal anti-inflammatory drops, which may be a confounding factor for reported IOP spike rates. Additionally, we observed that all patients on pre-existing topical glaucoma medication developed a significant IOP rise during the first 14 days postoperatively. Patients with history of glaucoma would be expected to have a higher chance of being steroid responder [26] and reduced aqueous drainage reserve, therefore are likely to be predisposed to pressure spikes after Densiron tamponade. While our data do not enable us to definitively determine the cause of IOP rise (including iatrogenic oil overfill, steroid response, or biological response to Densiron), the findings would suggest the need to closely monitor this high-risk patient population for IOP rise after Densiron fill.

### Inflammation and emulsification – anterior segment

Laboratory studies suggest that Densiron may cause a more pronounced inflammatory response in the eye compared to conventional silicone oils [27, 28]. This reaction may be caused by various pathways related to the added semifluorinated alkanes. Heavy silicone oils may induce greater mechanical injury due to gravity and increased lipophilicity which could increase retinal penetration [29]. Previously, heavy silicone oils have been found to be subject to chemical decomposition [30, 31], causing a separation of F6H8 followed by microemulsification which could then trigger type IV hypersensitivity reaction, leading to a vicious cycle as more proinflammatory cytokines and proteins accelerate emulsification. These observations point toward an increase in inflammation with increasing endotamponade duration. Semeraro et al. 2019 [27] showed that prostaglandin-E2 and interleukin-1-alpha levels significantly increased with the duration of the heavy silicone oil endotamponade. This increase of inflammation over time also explains the great difference in reported rates of inflammatory complications in clinical series, as the observation of inflammation strongly depended on the duration of endotamponade. Sandner et al. 2006 [32] and Stappler et al. 2009 [22] reported only mild anterior chamber reaction at 3 months, while Auriol et al. 2008 [25] showed a high rate of inflammatory reactions in eyes with a duration of the Densiron tamponade of > 6 months. In our study (with a mean tamponade duration of 4 months), there was little to no inflammatory complications in the anterior segment with only one case of intermittent anterior chamber cells. Thus, our findings are consistent with previous reports and emphasize tamponade duration as a major determinant of inflammatory reaction to Densiron. Also consistent with this observation is the low incidence of emulsified Densiron in this cohort, which is likely to depend on the tamponade duration.

### Visual loss after silicone oil removal

Unexplained visual loss after removal of lighter-than-water silicone oils (ROSO syndrome) has been reported in the literature with variable incidences ranging from 1–30% [33–36]. Several studies have investigated the potential cause of this worrying phenomenon and identified risk factors such as increased IOP and duration of silicone oil tamponade [33, 37], as well as younger patient age and macula-on retinal detachments [34, 36, 38]. Several hypotheses, which remain unproven, have been proposed to explain acute visual loss or central scotoma after silicone oil removal, including neuronal apoptosis [39, 40], phototoxicity [34, 41], changes in growth factor concentration [42] and direct oil-related cytotoxicity [43]. Interestingly, all previous articles on unexplained vision loss after silicone oil removal have focused on lighter-than-water silicone oils with only two reported cases of unexplained vision loss after the use of heavy silicone oil [44]. In our cohort, we did not encounter any case of unexplained vision loss following Densiron removal, even though a third of our study population was diagnosed as having ‘macula-on’ retinal status. While this question cannot be definitively answered with the limited sample size presented here, the absence of ROSO syndrome encountered following Densiron removal in our cohort is encouraging. This may be related to the properties of the oil itself, modest duration of Densiron tamponade, or a high proportion of complex cases which were macula-off thus masking symptom detection. Further studies using large datasets could help to compare the incidence of ROSO syndrome between different types of oil in matched cohorts, similar to recent work conducted by Tzoumas et al. which demonstrates superior anatomical and functional outcomes with Densiron tamponade in retinal detachments with inferior breaks and PVR compared with conventional oil [11].

### Inflammation – posterior segment

In previous studies, intraocular inflammation was mostly graded on clinical signs of anterior uveitis as inflammation in the posterior segment is difficult to quantify in silicone oil-filled eyes. Here we also present data on the prevalence of cystoid macular edema in a subset of patients with available OCTs acquired through Densiron. 20% of patients with Densiron tamponade developed CMO as an indicator of retinal inflammation. As OCTs are not routinely performed through oil at many institutions, data on OCT changes during Densiron tamponade is rare and inconsistent. In general, after a standard pars plana vitrectomy, around 5% of patients develop CMO [45]. Majid et al. [46] showed that in cases with severe emulsification during Densiron tamponade, a high rate of CMO of 37.5% can be observed. In contrast, Hostovsky et al. [47] noticed no signs of CMO in a cohort of 20 patients receiving Densiron tamponade for inferior retinal detachment with a similar tamponade duration but no documented signs of emulsification. Our study reports an occurrence of CMO within the previously mentioned range. Previously, perflourocarbons, a chemical closely related to the semifluorinated alkanes in Densiron, were shown to cause an inflammatory reaction similar to a foreign body response [48]. Additionally, low molecular weight components of silicone oils have been shown to diffuse from the oil into the ocular tissues [49], resulting in retinal toxicity [50]. Both pathways may be more pronounced in eyes with strong emulsification or active inflammation, such as PVR detachment. This therefore could explain the great variability in reported CMO rates. The manufacturer of Densiron 68 (Fluoron GmbH, Ulm, Germany) stated that no major modifications to the production process of Densiron were implemented since its market introduction.

As part of our evaluation of posterior segment toxicity in this study, we also evaluated subfoveal choroidal thickness which correlates well with subfoveal perfusion of the outer retina (i.e. photoreceptors) [51]. We found no significant change in choroidal thickness from the use of Densiron tamponade. This is the first ever study to assess choroidal thickness during heavy silicone oil tamponade. A recent meta-analysis [52] found three articles researching choroidal thickness in lighter-than-water silicone oil endotamponade. The published evidence suggests that longer durations of silicone oil endotamponade cause progressive thinning of the choroid. By comparing against fellow eyes, Karimi et al. [53] showed that this thinning is mostly associated with tamponade durations of 6 months or longer. Odrobina et al. [54] already reported an effect on choroidal thinning starting shortly after implantation in a cohort of 18 eyes. Mirza et al. [55] reports similar findings. The difference between Karimi et al. [53], Odrobina et al. [54], and Mirza et al. [55] might be explained by the different viscosities of the lighter-than-water silicone oils used as Karimi used high-viscosity 5700 cSt silicone oil while the other studies used 1000 cSt silicone oil. While our observation of no significant change in choroidal thickness with Densiron tamponade is based on a limited sample size, future study should be conducted to confirm this finding in a larger cohort as it may also provide mechanistic insight into the low rate of ROSO syndrome seen.

#### Lens-related complications

Finally, our study also evaluated lens-related complications. All phakic patients developed significant cataract until the time of Densiron removal. This is consistent with previous reports: Lappas et al. [56] showed that in a cohort of patients undergoing macular hole surgery with Densiron implantation with a similar tamponade duration 100% developed significant nuclear cataract that had to be removed at the time of Densiron removal. Li et al. [21] investigated the development of posterior capsule opacification (PCO) and found that approximately one quarter of eyes developed PCO which is in line with our observations.

### Limitations

Of course, this study has limitations. The retrospective design and the limited time of the follow-up are inherent limitations of retrospective cohort analyses. Additionally, regarding visual improvements it has to be taken into account that cataract surgery was often performed during the studied time span and likely contributes to the visual improvement. However, sparse data has been previously published on inferior PVR and Densiron 68. Further, this is a single center retrospective analysis with a limited case number, thus strong conclusions may not be drawn from the data presented here.

## Conclusions

In this study, we performed a comprehensive evaluation of potential complications associated with the use of Densiron 68 in the management of complex retinal detachments associated with PVR. The duration of endotamponade of around 4 months was well-tolerated, and little to no inflammatory complications were observed. Intraocular pressure rises were relatively common and usually occurred in the early postoperative period (< 14 days), which should be monitored. Unexplained visual loss as part of removal of silicone oil syndrome was not observed following Densiron removal in our limited cohort.

## Data Availability

Data is available from the corresponding author upon reasonable request.
